# Diagnostic value of routine ultrasonography combined with ultrasound elastography for papillary thyroid microcarcinoma: A protocol for systematic review and meta-analysis

**DOI:** 10.1097/MD.0000000000023905

**Published:** 2021-01-29

**Authors:** Yanfei Du, Yuyan Jiang, Shujuan Tang, Lijie Li

**Affiliations:** a Department of Ultrasound, Liaocheng People's Hospital; b Department of Ultrasound, Gaotang County People's Hospital, Liaocheng, Shandong Province, China.

**Keywords:** carcinoma, elasticity imaging techniques, papillary, thyroid neoplasms, ultrasound

## Abstract

**Background:**

Papillary thyroid microcarcinoma is easy to be missed because of its small focus, concealed incidence and lack of clinical features. Ultrasound examination is one of the main methods for the detection and diagnosis of papillary thyroid microcarcinoma. The detection rate of conventional ultrasound is not ideal. Combined ultrasound elastography can improve the detection rate, but there is lack of evidence-based evidence. The purpose of this study was to systematically evaluate the value of conventional ultrasound combined with ultrasound elastography in the diagnosis of papillary thyroid microcarcinoma.

**Methods:**

A systematic search was performed by retrieving on English databases (PubMed, Embase, Web of Science, the Cochrane Library) and Chinese databases (CNKI, Wanfang, Weipu (VIP), CBM). The retrieval time limit was from the establishment of the database to November 2020 and manually search for the conventional ultrasound combined with ultrasound elastography in the diagnosis of papillary thyroid microcarcinoma. Two researchers extracted and evaluated the quality of the data in the included study independently. A meta-analysis was performed using Meta Disc1.4 and RevMan5.3 software.

**Conclusions:**

This study will evaluate the accuracy and practicability of conventional ultrasound combined with ultrasonic elastography in the diagnosis of papillary thyroid microcarcinoma, and provide evidence-based basis for clinicians to choose the appropriate or best diagnostic method.

**Ethics and dissemination:**

The private information from individuals will not be published. This systematic review also will not involve endangering participant rights. Ethical approval is not required.

**OSF Registration number:**

DOI: 10.17605 / OSF.IO / V6HK7.

## 1 Introduction

In recent years, the incidence of thyroid cancer is increasing worldwide, which ranks first in the incidence of malignant tumors in the head and neck, with the incidence of papillary thyroid microcarcinoma (PTMC) increasing rapidly among thyroid cancer.^[1]^ Papillary thyroid microcarcinoma (PTMC) refers to thyroid cancer whose maximum diameter of the primary tumor ≤1 cm. According to the definition of WHO in 2004, PTMC is a subtype of papillary thyroid carcinoma, accounting for about 30% to 40% of all papillary thyroid carcinomas.^[2]^ Because PTMC patients usually have no clinical symptoms, about 76% of pathologically proved PTMC patients are missed in diagnosis. Even for experienced clinicians, nodules smaller than 2.0 cm may be missed in patients with thicker and shorter necks.^[3]^ Therefore, the diagnosis of PTMC mainly depends on imaging techniques and cytological examination.^[4]^ Because PTMC has small lesions, insidious onset, and often coexists with many other types of thyroid diseases,^[5]^ the detection rate of conventional two-dimensional ultrasound and other imaging methods, puncture biopsy and other detection methods are not ideal.^[6]^ Ultrasonic Elastography (UE) is a detection technology developed on the basis of conventional two-dimensional ultrasound, which can display the hardness information of tissues based on the color difference of images, and identify the nature of tumors by elastic classification and elastic parameters.^[7]^ It is also a non-invasive inspection method, which can clearly display the hardness of tissues.^[8]^

At present, there have been many studies on the diagnosis of PTMC by conventional ultrasound combined with ultrasound elastography,^[9–11]^ and it has been confirmed that the combination of the 2 has high diagnostic value for the differential diagnosis of PTMC. However, there is no reliable evidence-based evidence. The purpose of this systematic evaluation is to evaluate the accuracy and practicality of the combined use of the 2 imaging methods in the diagnosis of papillary thyroid microcarcinoma and to provide an evidence-based basis for clinicians.

## 2 Methods

### 2.1 Protocol register

The meta-analysis protocol has been drafted under the guidance of the preferred reporting items for systematic reviews and meta-analyses protocols (PRISMA-P),^[12]^ and it has been registered on open science framework (OSF)on November 26, 2020. (Registration number: DOI: 10.17605/ OSF.IO/V6HK7).

### 2.2 Ethics

Since this program does not involve patient recruitment and personal information collection, the approval of the Ethics Committee is not required.

### 2.3 Eligibility criteria

#### 2.3.1 Types of studies

We will collect case-control studies and cohort studies of ultrasound combined with ultrasound elastography in the diagnosis of thyroid microcarcinoma. And the language will be limited to Chinese and English.

#### 2.3.2 Object of studies

The subjects were PTMC patients, who were diagnosed by ultrasonography combined with elastography and confirmed by pathological examination as the gold standard. There were no limitations on nationality, race, age, gender, course of disease, etc.

#### 2.3.3 Types of diagnostic methods

The diagnostic method was conventional ultrasonography combined with elastography, and the gold standard was pathological results.

#### 2.3.4 Types of outcome indicators

Sensitivity (SEN), specificity (SPE), positive likelihood ratio (+LR), negative likelihood ratio (-LR), diagnostic odds ratio (DOR), summery receiver operating characteristic curve (SROC), area under curve (AUC) of conventional ultrasound combined with ultrasonic elastography in the diagnosis of PTMC.

### 2.4 Exclusion Criteria

1. Studies whose literature are abstract or data are incomplete, or whose data could not be gotten after contacting the author;
2. Conference summary, comments, abstracts, reviews, animal experiment, case reports, etc.
3. Studies without gold standard verification.
4. Studies published repeatedly should be the most recently published ones.

### 2.5 Search Strategy

“Conventional Ultrasound” (chuan tong chao sheng), “ultrasound elastography” (chao sheng tan xing cheng xiang), “papillary thyroid microcarcinoma” (jia zhuang xian wei xiao ru tou zhuang ai), “thyroid microcarcinoma” (jia zhuang xian wei xiao ai) were used for retrieval in Chinese databases, including CNKI, Wanfang Data Knowledge Service Platform, VIP Information Chinese Journal Service Platform, and China Biomedical Database. English retrieval words such as “Conventional Ultrasound”, “Ultrasound Elastography”, “Papillary Thyroid Microcarcinoma” were used for retrieval in English databases, including PubMed, EMBASE, Web of Science and the Cochrane Library. The retrieval time was from the establishment of the database to November 2020, and all the domestic and foreign literatures about the conventional ultrasound combined with ultrasound elastography in the diagnosis of papillary thyroid microcarcinoma were collected. Take PubMed as an example, and the retrieval strategy is shown in Table 1.

**Table 1:** Search strategy in PubMed database.

Number	Search terms
#1	Conventional Ultrasound [Title/Abstract]
#2	CUS [Title/Abstract]
#3	#1 OR #2
#4	ultrasonic elastography [Title/Abstract]
#5	UE [Title/Abstract]
#6	#4 OR #5
#7	pillary thyroid microcarcinoma [MeSH]
#8	Thyroid Neoplasms, Carcinoma, Papillary [Title/Abstract]
#9	pillary thyroid microcarcinoma [Title/Abstract]
#10	PTMC [Title/Abstract]
#11	#7 OR #8 OR #9 OR #10
#12	#3 AND #6 AND #11

### 2.6 Data screening and extraction

Two researchers independently extracted relevant data from each eligible study and recorded them through Excel 2013 according to the inclusion and exclusion criteria of the literature. The relevant data included:

1. Basic features of the included studies, including first author, published year, language, research country, number of test cases, imaging methods, gold standard, etc.
2. Key elements of bias risk assessment;
3. The outcome measurement index data of concern, such as true positive value, false positive value, true negative value, false negative value, etc.

**Figure 1:**
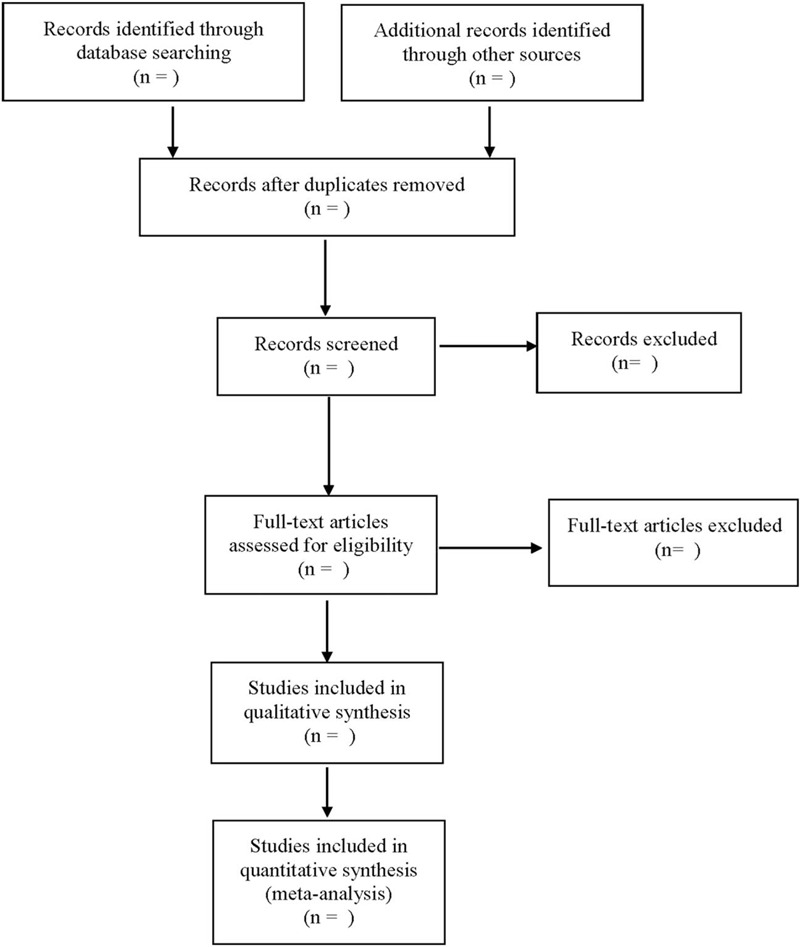
Flow diagram.

Disagreements are resolved through discussion or with the assistance of a third researcher. The literature screening process is shown in Figure 1.

### 2.7 Literature quality evaluation

The risk of bias in the included literature was independently evaluated by 2 researchers with reference to SQUADAS-2 quality evaluation criteria.^[13]^ QUADAS-2 defines methodological quality as a complex of bias risk and applicability. The evaluation of methodological quality of the included studies included 4 parts: patient selection, diagnostic method and gold standard of the test, test procedure and time interval. Each part includes several signal questions, which are answered by “yes”, “no” and “unclear”, and the methodological quality of the included studies is determined according to the answers to the signal questions. The 2 researchers completed the assessment and then cross-checked respectively. In case of any disagreement, discussion was required. If no agreement could be reached, a decision would be made in consultation with researchers from the third party.

### 2.8 Statistical analysis

#### 2.8.1 Data analysis and processing

Meta-analysis was performed using RevMan 5.3 software and Meta Disc 1.4 software. If (*P* > .10, *I*^*2*^ < 50%), it indicated low inter-study heterogeneity and the fixed-effect model was adopted to conduct a meta-analysis. If (*P* ≤ .1, *I*^*2*^>50%), it indicated higher inter-study heterogeneity and the random effects model was used for merging. Calculate the sensitivity (SEN), specificity (SPE), positive likelihood ratio (+LR), negative likelihood ratio(-LR), diagnostic odds ratio (DOR) and its 95% confidence interval (CI), draw summary receiver operating characteristic curve (SROC) and obtain area under curve (AUC). At the same time, the sensitivity analysis of the literature was excluded in sequence to evaluate the stability of the research results.

#### 2.8.2 Dealing with missing data

If there is missing data in the article, contact the author via email for additional information. If the author cannot be contacted, or the author has lost relevant data, descriptive analysis will be conducted instead of meta-analysis.

#### 2.8.3 Subgroup analysis

Subgroup analysis will be conducted according to different patient characteristics, indicators, reference tests and outcome indicators in this study.

#### 2.8.4 Assessment of publication bias

If there are more than 10 studies, the Deek funnel plot will be used to assess potential publication bias.^[14]^ What is more, Egger and Begg test would be used for the evaluation of publication bias.

#### 2.8.5 Grading the quality of evidence

We will use Grading of Recommendation Assessment, Development and Evaluation (GRADE) scoring method to grade the evidence of the outcome index.^[15]^ The evaluation includes bias risk, indirectness, inconsistency, inaccuracy and publication bias. The quality of evidence will be rated as high, medium, low or very low.

## 3 Discussion

The progress of PTMC is slow, but there is a corresponding risk of recurrence, distant metastasis and death, and young patients are more aggressive, especially in patients with lymph node metastasis.^[16]^ Therefore, early and timely diagnosis is very important. Ultrasound examination is convenient, efficient and cheap, and its detection rate of thyroid diseases is significantly better than CT, MRI and radionuclide examination, etc. Therefore, ultrasound is the preferred detection method for thyroid diseases.^[17]^

In conventional two-dimensional ultrasound, the benign and malignant features of thyroid microlesion may not be obvious. That's because when the infiltration scope of thyroid microlesions is small or the basement membrane has not broken through the surrounding infiltration, resulting in secondary changes of surrounding tissues, conventional ultrasound often shows regular morphology and clear boundaries, and there are no obvious malignant features, which is not conducive to diagnosis. However, ultrasound elastography is mainly diagnosed by comprehensive analysis of tissue hardness, which is different from the mechanism of conventional two-dimensional ultrasound imaging, so it can better find thyroid microlesions and make more accurate differential diagnosis of benign and malignant tumors,^[18]^ especially for papillary carcinoma. However, the elastic coefficients of all kinds of tissues overlap, so misdiagnosis may occur in the process of detection.^[19]^ Studies have found that thyroid cancer diagnosed by ultrasound elastography alone may be misdiagnosed,^[20]^ while conventional ultrasound can supplement other diagnostic basis, such as irregular shape, low echo, unclear echo boundary, relatively large aspect ratio (≥1), microcalcification and lack of halo ring, etc.

Conventional ultrasound combined with ultrasound elastography has its own advantages in the diagnosis of papillary thyroid microcarcinoma, but there is no consistent conclusion on the accuracy of its diagnosis, and currently there is no systematic review on this topic. On the basis of summarizing the current research, this study will systematically and comprehensively evaluate the accuracy, sensitivity and specificity of conventional ultrasound combined with ultrasound elastography in the diagnosis of papillary thyroid microcarcinoma.

However, this systematic review has certain limitations: patients in different studies have different course of disease, age, and degree of disease; conventional ultrasound and ultrasound elastography diagnostic criteria for PTMC in different studies may be inconsistent; differences in diagnostic equipment and qualifications of diagnostic physicians may lead to certain clinical heterogeneity. In addition, due to the limitation of language ability, we only search English and Chinese literature and may ignore studies or reports in other languages.

## Author contributions

**Data collection:** Yuyan Jiang and Shujuan Tang.

**Funding acquisition:** Lijie Li.

**Investigation:** Yanfei Du, Shujuan Tang.

**Literature retrieval:** Yanfei Du and Shujuan Tang

**Resources:** Yuyan Jiang, Shujuan Tang.

**Software:** Yuyan Jiang.

**Supervision:** Shujuan Tang.

**Writing – original draft:** Yanfei Du, Yuyan Jiang.

**Writing – review & editing:** Yanfei Du, Lijie Li.

## Abbreviations

Abbreviations: +LR = positive likelihood ratio, AUC = area under curve, DOR = diagnostic odds ratio, -LR = negative likelihood ratio, PRISMA-P = preferred reporting items for systematic reviews and meta-analyses protocols, PTMC = papillary thyroid microcarcinoma, SEN = sensitivity, SPE = specificity, SROC = summery receiver operating characteristic curve, UE = ultrasonic elastography.
